# Serum Biomarkers for Chemotherapy Cardiotoxicity Risk Detection of Breast Cancer Patients

**DOI:** 10.31557/APJCP.2021.22.10.3355

**Published:** 2021-10

**Authors:** Diya Hasan, Yazan Ismail, Ahmad Al Tibi, Safaa A AL-Zeidaneen, Mohanad Ode, George J Burghel, Iyad Natsheh, Amid Abdelnour

**Affiliations:** 1 *Al-Balqa Applied University, Zarqa College, Department of Allied Medical Sciences, Zarqa, Jordan. *; 2 *Higher Colleges of Technology, Faculty of Health Sciences, Abu Dhabi, United Arab Emirates. *; 3 *Biolab Diagnostic Laboratories, Amman, Jordan. *; 4 *Hashemite University, Faculty of Pharmaceutical Sciences, Department of Clinical Pharmacy and Pharmacy Practice, Zarqa, Jordan. *; 5 *Manchester University NHS Foundation Trust, Manchester Centre for Genomics Medicine, Manchester, UK. *

**Keywords:** Hs-CRP, Apo-B- chemotherapy, cardiotoxicity, breast cancer

## Abstract

**Objective:**

This study aimed to investigate level fluctuations of serum biomarkers that are associated with cardiotoxicity risk, such as high-sensitivity C-reactive protein (hs-CRP) and apolipoprotein-B (Apo-B) in response to chemotherapy treatment for breast cancer.

**Method:**

The serum levels of hs-CRP and Apo-B were evaluated in 56 breast cancer patients with main inclusion criteria: HER2 negative and who received adjuvant chemotherapy AC [A: Adriamycin, C: Cyclophosphamide] or AC→T [A: Adriamycin, C: Cyclophosphamide, T: Taxane] regimes at early II (n = 26) and late IV (n = 30) clinical stages by using particle enhanced turbidimetric assay.

**Results:**

The results of this study suggest that a high level of pre-treatment hs-CRP is a good prognostic marker in comparison to Apo-B. Moreover, the AC-T chemotherapy regime treatment in both early and late stages exhibited a significantly higher level of hs-CRP compared to that in the AC regime. Hs-CRP was significantly elevated in the early stage in comparison to the late stage among cancer patients, meanwhile Apo-B behaved inversely. Furthermore, the results showed that hs-CRP levels were significantly higher in late-stage cancer patients compared with those in early-stage in both chemotherapy regimens groups. On the other hand, Apo-B showed no significant differences.

**Conclusion:**

Monitoring hs-CRP level changes in comparison to Apo-B can be used to assist the side effect risk difference among different chemotherapy regimens, and staging reflecting a positive correlation between them more notable in the late stage.

## Introduction

Breast cancer is the most common cancer that affects women in Jordan. It has a high mortality rate, as it commonly occurs after lung and colorectal cancers (Abdel-Razeq et al., 2020). In therapeutic management, there are many therapeutics protocols that can be followed depending on various factors determined by the supervised physician. Chemotherapy regimens usually consist of a combination of drugs that are given in a number of cycles at a set period of time, depending on the effectiveness of clinical trials for a specific regimen. There are many types of chemotherapy used to treat breast cancer, among them are alkylating agents such as Cyclophosphamide (Cytoxan), anthracycline antibiotics, and antineoplastics such as Doxorubicin hydrochloride (Adriamycin), and antimicrotubule and Taxane derivatives such as paclitaxel (Taxol) and docetaxel (Taxotere). Chemotherapy regimen could be given as AC [A: Adriamycin, C: Cyclophosphamide] or AC→T [A: Adriamycin, C: Cyclophosphamide, T: Taxane] (society, 2019) (Drugs, 2021).

Cardiotoxicity risk associated with chemotherapeutic agents is a common complication and remains a major limitation that strongly affects patients’ quality of life and survival. Several studies had indicated that patients with no history of cardiovascular disease may develop symptoms of heart failure associated with the accumulation of received chemotherapy dose. This has caused a change in chemotherapy regimens and reduction in doses leading to reduced efficiency (Jensen, 2006). The issue of chemotherapy-associated cardiotoxicity is continuously rising among cancer patients (Herbst et al., 2006; Hong et al., 2010).

The most studied chemotherapy drugs related to unfavorable cardiac incidents are anthracyclines (Doxorubicin), in addition to Taxanes. Doxorubicin affects cardiac myocytes by inducing necrosis and apoptosis causing myocardial fibrosis, which is irreversible (Sawyer et al., 2010; Youn et al., 2005). The inhibition of DNA, RNA, and protein synthesis (Lim et al., 2004), and transcription factors that control cardiac genes expression (Takahashi et al., 1998) (Lebrecht et al., 2005), adjust adenylyl and adrenergic cyclase activity (Mordente et al., 2009) and interfere calcium homeostasis (Dodd et al., 1993). 

Several cardiovascular side effects were associated with chemotherapy treatment, such as hypertension, acute heart failure, arrhythmias, myocardial ischemia, and late-onset ventricular dysfunction (Eschenhagen et al., 2011; Shan et al., 1996). The cardiac toxicity ranged from acute presentation during treatment to chronic cases persisting months after treatment completion. The most significant clinical outcome is weakened left ventricular ejection fraction (LVEF) causing symptomatic heart failure (Dolci et al., 2008; Germanakis et al., 2008) (Mavinkurve-Groothuis et al., 2008). 

Detection of cardiotoxicity is achieved by the evaluation of LVEF using echocardiography, magnetic resonance imaging (MRI), and radionuclide ventriculography (a resource-intensive test which depends on the operator) at the beginning of the therapy course, after half of the dose has been given to the patient, before each upcoming dose, and 12, 24, and 48 weeks after course completion (Plana et al., 2014). Because a certain amount of myocardial damage is required to induce a decrease of LVEF, the observation of cardiac toxicity can be delayed causing irreversible cardiac damage and a delay with heart failure therapy with no total recovery. Only 42% of patients with cardiac toxicity were able to reach full cardiac healing, regardless of optimal heart failure therapy (Cardinale et al., 2010). 

Measurement of cardiovascular-specific biomarkers can be used as a diagnostic tool for early identification of cardiotoxicity risk before it becomes clinically apparent. This approach is less expensive and less invasive compared with echocardiography, and can be easily repeated without patient irradiation. Besides, interpretation of the results is objective, which reduces the risks of inter-observer variability (Cardinale and Sandri, 2010).

C-reactive protein (CRP) is an acute-phase protein that is expressed during inflammatory response. The increase of its level is predictive of diastolic dysfunction and decreased LVEF in correlation with other cardio clinical outcomes, such as myocardial infarction, congestive heart failure, and stable coronary artery disease (Arruda-Olson et al., 2010; Arroyo-Espliguero et al., 2009; Windram et al., 2007). The measurement of high-sensitivity C-reactive protein (hs-CRP) using a high-sensitivity (hs) assay had a predicted impaired LVEF with 92.9% sensitivity and 45.7% specificity in breast cancer patients (Onitilo et al., 2012).

Through mechanisms that are not fully understood, high levels of apolipoprotein-B (Apo-B), correlated with high low-density lipoprotein (LDL) levels, are the major reasons causing vascular disease (atherosclerosis), leading to life-threatening complications, such as heart disease and stroke (Shapiro and Fazio, 2017). Chemotherapy was notably found to change plasma lipid and apolipoprotein levels in breast cancer patients, as Apo-B levels significantly increased (Sharma et al., 2016). Several lipid parameter levels, including Apo-B and LDL, were increased in breast cancer patients by comparing the first cycle to the last cycle of chemotherapy (Li et al., 2018).

Therefore, the present study aimed to evaluate the hs-CRP and Apo-B serum level variations in response to different chemotherapy regimens and clinical stages to be used in assessing cardiotoxicity risk during treatment.

## Materials and Methods

*Patient’s cohort*

This study included a total of 56 female breast cancer patients with main inclusion criteria: 1) HER2-negative as analyzed by immunohistostaining, and 2) received adjuvant chemotherapy regimen AC [A: Adriamycin, C: Cyclophosphamide] or AC→T [A: Adriamycin, C: Cyclophosphamide, T: Taxane]. The patients’ characteristics are listed in Table 1. Out of 56 cases, 26 patients were classified as clinical stage II and 30 patients were classified as clinical stage IV. Based on the TNM (Tumor, Nodes and Metastases) and American Joint Committee on Cancer (AJCC) staging system, stage ≤II was classified as early-stage and stage >II was classified as late-stage (Edge and Compton, 2010). The median age of the advanced disease group was 48 (43 – 53) years, while of the early-stage group was 50 (45 – 55) years. There was no significant difference (p=0.232) between the two groups.

This study was conducted in agreement with the ethical standards of human research. The authorization of the local medical ethics committee of Al Bashir Hospital Amman, Jordan (approval # 3345) was acquired for all groups. Venous blood samples for hs-CRP and Apo-B diagnosis were collected from patients after three months of chemotherapy treatment (the 4^th^ chemotherapy cycle). Samples were processed at the department of biochemistry, Biolab diagnostic laboratories Amman, Jordan. The serum was separated by centrifugation (2500 rpm for 10 min); aliquots were stored at -20°C for later assay. The serum levels of hs-CRP and Apo-B were measured using particle enhanced turbidimetric assay for hs-CRP and immune turbidimetric assay for Apo-B (Cobas 6000 Roche Hitachi, Switzerland). Marker assays were done using commercial kits hs-CRP (Cardiac C - reactive protein (Latex) High Sensitive, Cobas) and Apo-B (Tina-quant Apolipoprotein B ver.2 Cobas). A cut-off point of <5.0 mg/L (hs-CRP) and 60-117 mg/dl (Apo-B) were used as suggested by Roche diagnostic. The hs-CRP and Apo-B measurements data of 20 healthy females (control negative) and 16 pre-chemotherapy breast cancer female patients (control positive) were obtained from Bio-lab laboratories.

*Primary chemotherapy*

Out of 56 breast cancer patients, 14 and 15 patients classified as stage II and IV, respectively, were treated with four cycles of AC (Adriamycin 50 mg/m^2^ and Cyclophosphamide 1,000 mg/m^2^ ) at day one and repeated every 21 days. Meanwhile, the other group consisted of 12 and 15 patients classified as stage II and IV, respectively, who received the previous chemotherapy regimen AC followed by four cycles of T (Taxotere 80 mg/m^2^) every 21 days.

*Statistical analysis*

Descriptive results were presented by proportions for dichotomous variables, and median values with the interquartile ranges for continuous variables. Mann-Whitney U test was done to determine the differences in enzymes level between groups. The Chi-square test was used to determine whether the difference in proportions was statistically significant. The odds ratio was used to determine the association where possible. 

## Results

This study was designed to measure hs-CRP and Apo-B levels to evaluate their significance and correlation with different chemotherapy regimens at different clinical stages in women with breast cancer from Jordan. 

**Figure 1:**
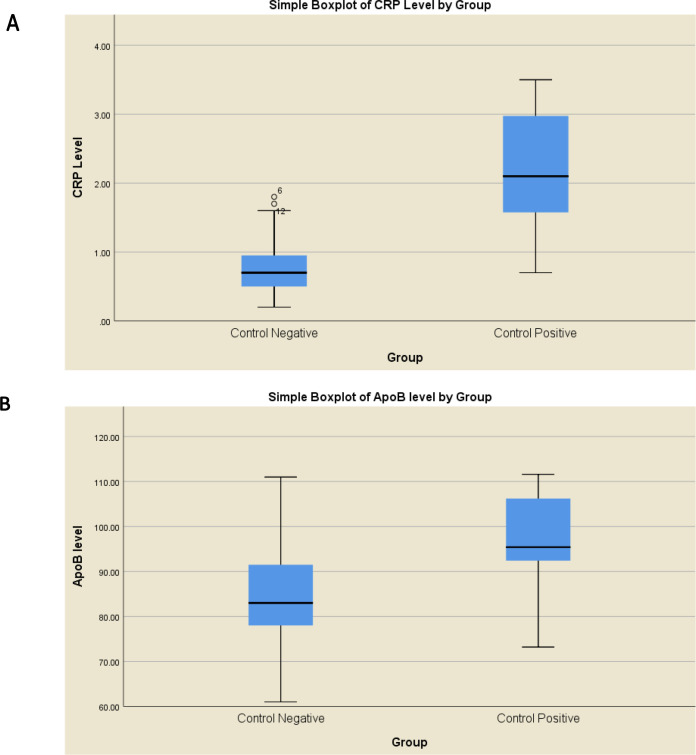
Serum Level Comparison between Patient Groups before Chemotherapy (positive control) and Non-cancer Group (negative control) of (A) CRP (B) ApoB

The levels of hs-CRP and Apo-B were measured in all samples using enzyme-linked immunosorbent assay (ELISA). The statistical analysis showed the hs-CRP distribution of differences between both groups. Hs-CRP for control positive patients (mean rank = 29.3, Median =2.45) were statistically significantly higher than those control negative cases (mean rank = 11.7, Median =0.7), U = 296, z = 4.337, p < 0.001 (Figure 1A). Distributions of the Apo-B for both groups were also different. Apo-B levels of positive control group cases (mean rank =24.06, Median =95.4) were statistically significantly higher than those of negative control group cases (mean rank = 14.05, Median =83.0), U = 249, z = 2.834, p = 0.004 (Figure 1B). The presented data demonstrated hs-CRP and Apo-B serum levels discrimination between both groups. However, only a small percentage of breast cancer patients had a higher level of Apo-B above the cut-off point (Figure 1B) compared with hs-CRP (Figure 1A), confirming the low sensitivity of this marker in breast cancer diagnosis.

Overall, elevated positive serum levels found in clinical stages II and IV were identified as follows: hs-CRP [II AC 7/14 (50%)], [II AC-T 7/12 (58.3%)], [IV AC 9/15 (60%)] and [IV AC-T 11/15 (73.3%)] of the breast cancer cases using cut off point <5.0 mg/L (hs-CRP). In total 14/ 26 (53.8%) of stage II and 20/30 (66.6%) of stage IV patients had higher levels of hs-CRP than the cut-off point. Apo-B [II AC 9/14 (64.2 %)], [II AC-T 2/12 (16.6 %)], [IV AC 9/15 (60 %)] and [IV AC-T 2/15 (13.3 %)] of breast cancer cases using a cut-off point 60-117 mg/dl (Apo-B). In total, 11/26 (42.3 %) of stage II and 11/30 (36.6 %) of stage IV patients had higher levels of Apo-B than the cut-off point (Table 2). In this study, the late-stage IV patients group exhibited a higher level of hs-CRP but not Apo-B compared with early-stage II patients for both chemotherapy regimens (AC and AC-T). Statistically significant differences between proportions of elevated CRP and Apo-B levels were demonstrated in Stage IV AC-T (X2(1)=10.99,P=0.001 Odds Ratio [OR]= 17.875; 95% CI: 2.73 - 116.88, P=0.003) and Stage IV total (X^2^ (1)=5.41, P=0.02 OR =3.455; 95% CI:1.195 - 9.990, P=0.022). Accordingly, serum levels of hs-CRP would be more efficient as an indicator for cardiotoxicity risk in advanced tumors than early diagnosis as compared with Apo-B, especially for the AC-T regimen (Table 2).

**Figure 2:**
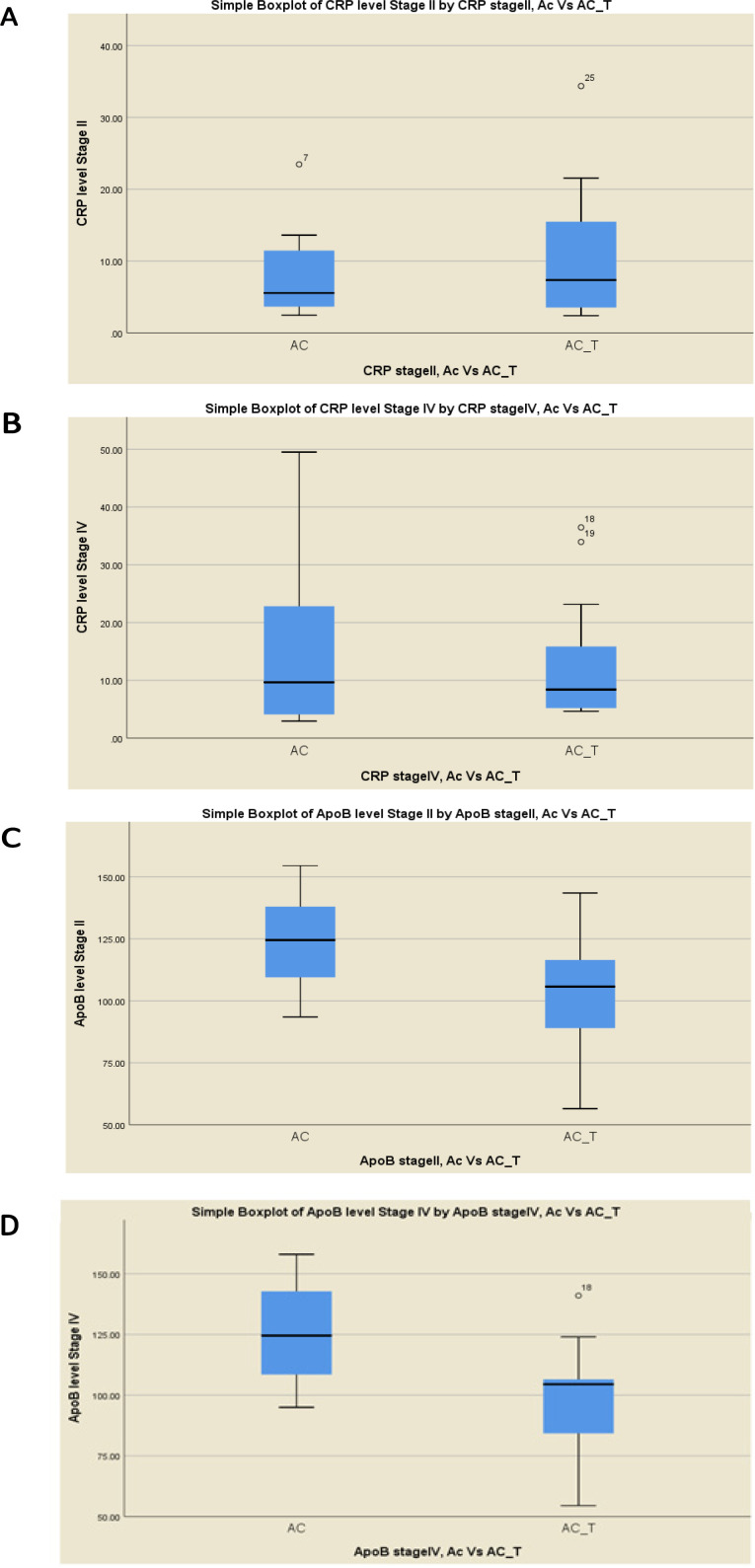
Serum Markers Level Response in Correlation with Chemotherapy Regime Type AC or AC-T in both Clinical Stages (A) CRP Level in Stage II (B) in Stage IV, (C) ApoB Level in Stage II (D) in Stage IV

The (AC and AC-T) chemotherapy regimens in both clinical stages II and IV were compared to check if markers of serum level response are connected with the type of chemotherapy regime. There was a correlation between change in hs-CRP levels and response to treatment. The elevation of the hs-CRP level was higher in the AC-T regimen patients’ group as compared to that in AC in early stage II; median levels were 7.35 (3.46 – 17.61) and 5.5 (3.48 – 11.82), respectively. However, the sample size (n=12 and n=14) did not permit a statistically significant difference (Figure 2A). The same elevation was also noticed in late stage IV, median CRP levels were 8.4 (4.95 – 16.5) for AC-T and 9.65 (4.03 – 23.21) (Figure 2B). Despite this, the elevation was higher in stage IV than that in stage II but was not statistically significant. Meanwhile, Apo-B response was different from that of hs-CRP, where the level of Apo-B was significantly lower in the AC-T regimen group as compared to that in AC in both stages. In the early stage, median Apo-B levels for AC-T were 86.75 (105.75 - 116.5) and 124.5 (106.5 – 139.00) for AC, P=0.023 (Figure 2C). For the late stage, median Apo-B levels for AC-T 104.5 (83.0- 106.5) and for AC 124.5 (106.5 – 143.0), P=0.002 (Figure 2D).

**Figure 3:**
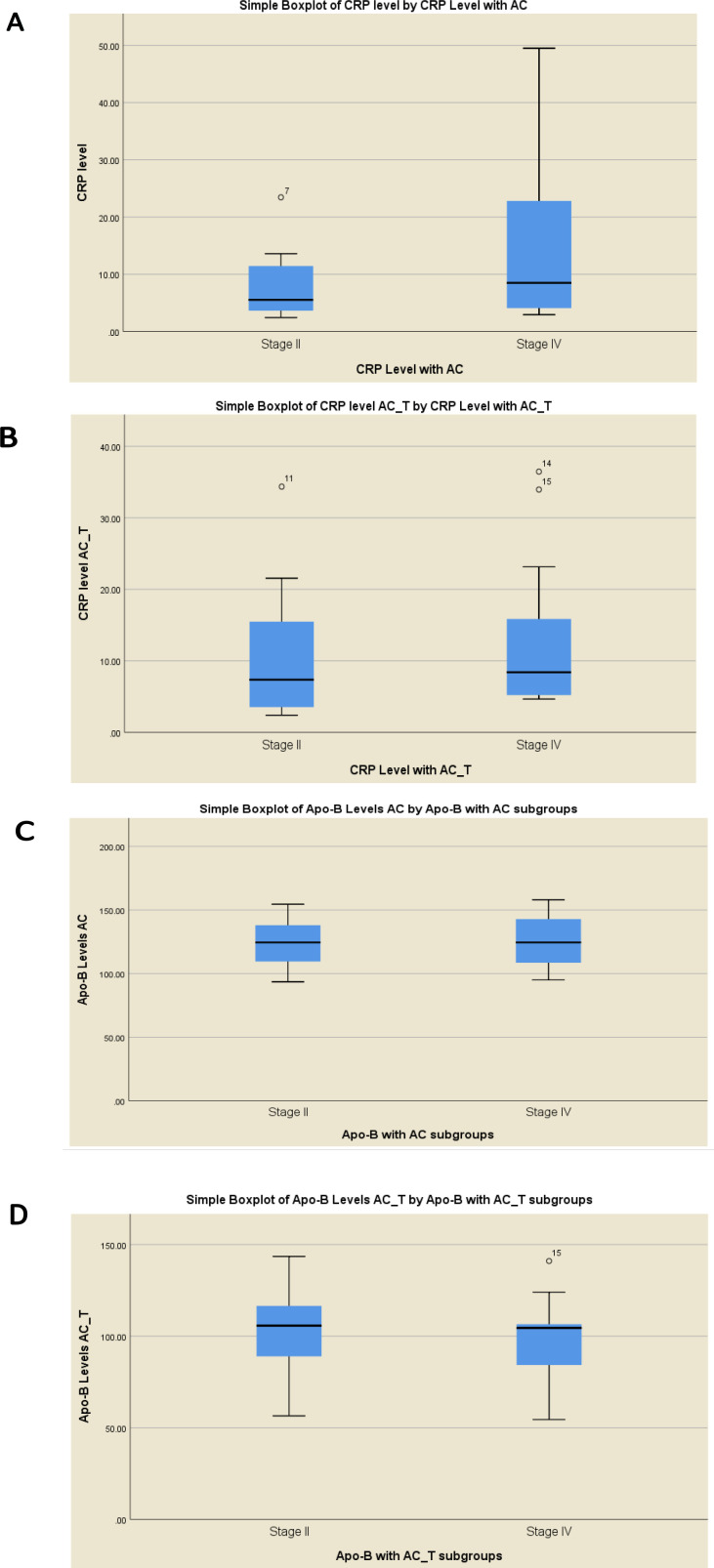
Serum Markers Level Response in Correlation with Clinical Stages II and IV. (A) CRP Level in AC (B) in AC-T, (C) ApoB Level in AC (D) in AC-T

The elevation of hs-CRP and Apo-B is related to chemotherapy regimens (AC or AC-T) treatment and is associated with staging progress of breast cancer patients. Interestingly, a correlation was found between hs-CRP level and was both used as chemotherapy regimens in each stage. Elevation of hs-CRP level was noted in the late stage as compared to that in the early stage group in both chemotherapy regimens, as it was greater in the AC regimen as compared to that in the AC-T regiment but was not statistically significant. For the AC regimen median CRP levels for stage II was 5.5 (3.5 – 11.8) and that for stage IV was 8.5 (3.9 -23.6), P=0.08 (Figure 3A). For the AC-T regimen, median CRP levels for stage II were 7.4 (3.5 – 17.6) and for stage IV, it was 8.4 (4.9 – 16.5), P>0.05 (Figure 3B). Meanwhile, the mean levels of Apo-B showed no significant differences between the two stages compared to one another for both chemotherapy regimens, AC median Apo-B levels for AC stage II was 124.5 (106.5 – 139.0) and for stage IV it was 125.5 (106.5 -143.0), P>0.05 (Figure 3 C) and for AC-T median Apo-B levels for AC Stage II was 124.5 (106.5 – 139.0) and for stage IV it was 125.5 (106.5 -143.0), P>0.05 (Figure 3D).

**Tabel 1:** Patient Characteristics and Treatment Modality

Patient parameter	n (%)
Age (years)	Median 49
	Range 43-55
Gender	Female
Cilinical stage	
Stage I	26 (46.4 %)
Stage IV	30 (53.5 %)
Chemotherapy regime	
AC × 4	29 (51.7%)
AC × 4 followed by T × 4	27 (48.2%)
Histological type	IDC
HER2 receptor	-Ve

**Table 2:** Serum Levels of CRP and ApoB in Clinical Stages II and IV for both Chemotherapy Regimens (AC and AC-T) P < 0.001, P < 0.01

hs-CRP			Apo -B			P value of differences between CRP and Apo B
II AC	7/14	50%	II AC	9/14	64.20%	0.445
II AC-T	7/12	58.30%	II AC-T	2/12	16.60%	0.089
IV AC	9/15	60%	IV AC	9/15	60%	1
IV AC-T	11/15	73.30%	IV AC-T	2/15	13.30%	0.001*
Total CRP			Total Apo-B			
stage II	14/26	53.80%	stage II	11/26	42.30%	0.405
stage IV	20/30	66.60%	stage IV	11/30	36.60%	0.02*

## Discussion

Anticancer drugs are correlated with cardiotoxicity, hence it is important to monitor patients receiving chemotherapy. Biomarkers may assist in distinguishing patients at high risk before initial therapy, in addition to during follow-up.

In the present study, it was found that both hs-CRP levels and Apo-B markers were significantly elevated in breast cancer patients at the time of diagnosis, in comparison with healthy controls with a preference for hs-CRP over Apo-B. Various epidemiological studies have revealed a positive connection between hs-CRP level and breast cancer risk (Siegel et al., 2015; Allin et al., 2011). Inflammation and cancer were linked to each other, as it stimulates tumor growth and progression while the tumor presents an inflammatory microenvironment (Allin et al., 2009). Increased serum CRP levels were also correlated with the progression of different types of malignancies including breast cancer (Roxburgh and McMillan, 2010). Moreover, elevated levels of serum CRP can be associated with heart disease (Wang et al., 2017; Takeuchi et al., 2017). The epidemiological proof for the association between cancer and apolipoproteins has not been extensively explored and the outcomes have been contradictory. Apo-A1 increased levels were linked with higher rates of breast cancer cases, and inversely the rate of breast cancer was less among women with high Apo-B (Borgquist et al., 2016). The results suggest that high increase of CRP and moderate increase of Apo-B serum levels elevation in the pre-treatment group were indicators of good and weak prognostic factors, respectively. 

The results reveal that hs-CRP and Apo-B levels were higher among post-chemotherapy patients as compared to pre-chemotherapy ones for both investigated AC and AC-T chemotherapy regimens in early and late stages. Hs-CRP level was shown to increase lung and breast cancer in patients after chemotherapy. Studies have shown alternations in the lipid profile of breast cancer patients, including Apo-B, before and after multi-agent chemotherapy treatments (Sharma et al., 2016). Some hypotheses suggest that chemotherapy could result in endothelial dysfunction, leading to cytokine changes, consequently causing lipid development (Vehmanen et al., 2004; Kim et al., 2006). 

In the present study, serum hs-CRP level results were significantly different among studied chemotherapy regimens. Data analysis indicated that elevated hs-CRP was more observed in the AC-T regimen as compared to AC one in both early and late stages, whereas Apo-B had behaved conversely. These data support an inverse association between hs-CRP and Apo-B levels in response to chemotherapeutic regimens. The use of AC alone or followed with other chemotherapeutic agents is common for breast cancer treatment (Early Breast Cancer Trialists’ Collaborative, 2005). Their use has been restricted due to notable toxic effects on the heart including cardiac cellular injury induction (Shan et al., 1996). Several factors can increase AC-induced cardiotoxicity risk such as a cumulative dose, age, and gender (Smith et al., 2010). Hs-CRP level is elevated in the circulation in response to tissue damage, acute inflammation, and infection. This elevation is correlated to tissue damage intensity. 

In the current study, the late stage IV patient group showed a higher hs-CRP level as compared to that in the early stage II in response to both studied chemotherapy regimens, indicating a significant positive correlation between hs-CRP and clinical stages. On other hand, Apo-B level showed no significant differences between clinical stages, which supports that hs-CRP is more sensitive than Apo-B. Studies have shown that serum hs-CRP levels are associated with tumor stages in several cancers including cervical cancer (He et al., 2018). Furthermore, hs-CRP consistently raised during treatment could indicate an unfavorable prognosis for nasopharyngeal carcinoma (Chen et al., 2019). The elevation of hs-CRP concentrations was associated with clinically related poorer survival in each stage of colon cancer (Kersten et al., 2013). 

From a clinical perspective, the outcomes of the present study are important since one of the main problems of breast cancer patients in Jordan was the following from the early stage to the late stage, and monitoring who are at risk for developing heart disease caused by chemotherapy. Therefore, establishing sensitive and dependable serum biomarkers selection for monitoring during diagnosis and treatment can decrease the rate of mortality and enhance treatment quality.

In conclusion, this study recommends that higher levels of hs-CRP would be a reliable prognostic marker as compared to Apo-B. Significant changes of hs-CRP in this study suggest that cardiotoxicity risk may happen during courses of treatment and differ among used regimens. Finally, a dose-effect correlation between elevated hs-CRP levels and prognosis shows that hs-CRP could be valuable in predicting prognosis in advanced cancer patients.

## Author Contribution Statement

Dr. Diya Hasan (corresponding author). Study design, study management, manuscript writing, and data analysis. Dr. Yazan Ismail. Study design, collecting samples. Ahmad Al Tibe. Samples analysis, data collection. Dr. Safaa A AL-Zeidaneen. Study design, samples collection. Dr. Mohanad Ode. Data analysis, statistical analysis. Dr. George J Burghel. Manuscript writing, data analysis. Dr. Iyad Natsheh. Manuscript reviewing, data analysis. Dr. Amid Abdelnour. Sample analysis, study management.
